# Implication of noise exposure on hearing with emphasis to hOGG1 and GPx-1 polymorphisms and HO-1 protein among textile workers

**DOI:** 10.1007/s11356-023-31590-6

**Published:** 2023-12-26

**Authors:** Mona Mohamed Taha, Lamia Samir Ellaithy, Nermeeen Said Abd El-Aziz, Heba Mahdy-Abdallah, Mona Adel Helmy

**Affiliations:** Department of Environmental and Occupational Medicine, Environment and Climate Change Research Institute, National Research Centre, Giza, Egypt

**Keywords:** Hearing impairment, Noise, Audiometric notches, hOGG1, GPx genes, HO-1 protein

## Abstract

Noise exposure is a health hazard in the textile industry. In cochlear hair cells, DNA damage caused by 8-oxoguanine (8-oxo G) can result in noise-induced hearing loss. Human 8-hydroxyguanine glycosylase (hOGG1) is a DNA repair enzyme that excises (8-oxo G) in the DNA and repairs DNA damage. Glutathione peroxidase-1 (GPx) is a crucial antioxidant enzyme that aids in limiting cochlear damages. Heme oxygenase-1 (HO-1) is a stress-inducible protein with a high fold change in the hair cells of the cochlea. The study aimed to investigate the association of either hOGG1 and GPx-1 polymorphisms with audiometric notches and HO-1 protein among textile workers. hOGG1 and GPx genotypes were analyzed by PCR–RFLP, and HO-1 levels were measured by ELISA in 115 male textile workers. Blood pressure and audiogram were performed. Results recorded the relation between audiometric notches and ear complaints among workers. Older age workers showed audiometric notches at > 25 dB with a significant decrease in HO-1 levels and higher levels in workers with normal audiogram. Ser/Cys genotype of hOGG1 gene was associated with age and work duration while CC genotype of GPx is associated with HO-1 levels and diastolic pressure. Ser/Cys genotype of hOGG1 gene was associated with age while Cys/Cys genotype was associated with work duration among workers. CC genotype of GPx gene was associated with higher HO-1 levels and TT genotype was associated with high diastolic pressure. Finally, hearing impairment was dependent on the duration of exposure to noise, older age, and the presence of heterozygote TC genotype of GPx gene among textile workers.

## Introduction

Long-term exposure to noise may lead to damage in the peripheral auditory system. This includes cochlea hair cells structure as well as cilia, tectorial membrane, and supporting cells (Wang et al. 2022), with the most in terms of hitting the external layer of hair cells and the Corti’s organ and also spiral ganglion might undergo degenerative changes. Moreover, exposure to noise may lead to a damage in the central auditory system that is mainly shown in the nucleus of the cochlea, medial geniculate body, olivary nucleus, inferior colliculus, auditory cortex, and hippocampus (Chen et al. 2022).

The notch was shown when the threshold at the notch frequency (3000, 4000, or 6000 Hz) minus the 2000 Hz threshold and the threshold at the notch frequency minus the 8000 Hz threshold were both greater than or equal to 10 dB (Wilson and McArdle 2013; Taha et al. 2022).

Tinnitus is a sound perception in the ears or head without any external stimulus. Moreover, it is generally recorded in 10–15% of the adult population with an increase in prevalence with age. Furthermore, tinnitus did not only vary in terms of its location and sound perception but also varied in terms of distress (Assouly et al. 2022). Its definition was a frequent disorder that affects all strata of populations and mainly it represents a health concern (Taha et al. 2022). Usually, hearing loss is a result of the death of hair cells (HCs) found in the inner ear. These hair cells constitute auditory and balance sensory cells that allow the conversion of mechanical stimuli to neural signals (WenWei et al. 2018). These Hcs are susceptible to different stressors including age, gene mutations, and noise trauma as well as treatment with ototoxic drugs (Yang et al. 2022).

A highly noisy work environment was shown in the textile manufacturing industry and that is because of the weaving machines with a noise intensity which exceeded the threshold value. This noise could cause complaints leading to several hearing disorders. Many factors can affect hearing like age, work period, and using equipment for ear protection (Damayanti et al. 2022). Also, hearing damage at different levels was observed in many individuals who were exposed to a similar noisy environment, suggesting the definite role of genetic predisposing (interaction of genetic and environmental factors) (Bahaloo et al. 2020; Taha et al. 2022).

While noise-induced hearing loss (NIHL) represents a typical type of hearing loss that is attributed to both genetic and environmental factors. Long-term exposure to noise was shown to be a NIHL prominent environmental factor. Other studies demonstrated that not every worker exposed to the same noise level should develop NIHL and also great variation in the severity of NIHL was shown. The hearing threshold of workers in a textile factory was detected by Taylor et al. (1965) who indicated that individuals who worked with similar length of service might show varying hearing thresholds in the range from 10 to 70 dB (Chen et al. 2022).

In the base excision repair pathway (BER), human 8-hydroxyguanine glycosylase (hOGG1) was defined as a DNA repair enzyme. Its main function is the recognition and excision of 8-oxo G in double-stranded DNA for repairing damaged DNA. 8-oxoguanine (8-oxoG) is able to lead to DNA damage to cochlear hair cells. This damage can develop NIHL essentially (Chen et al. 2022). The previous study conducted by Shen et al. (2014) investigated whether a relationship was demonstrated between hOGG1 Ser326Cys gene polymorphism (rs1052133) and its susceptibility to high-frequency hearing loss.

Several defense mechanisms (as intracellular enzymes) role is to control the oxidative stress attacking the cellular system (Irfan et al. 2016). Glutathione peroxidase 1 (GPX1) is an antioxidant enzyme that has a crucial role in preventing intra-cellular harmful accumulation of hydrogen peroxide. GPX1 can contribute in limiting cochlear damages that are in association with either age or acoustic overexposure (Wang et al. 2022). The GPX1 gene located at chromosome 3 (p21 locus) controlled GPX1 enzyme activity. The 5-UTR of GPX1 contains a single nucleotide C/T polymorphism (rs1800668) located at the position ch3:49395757. Regarding the activity of GPX1 enzyme, this C/T polymorphism is of great importance. The CC genotype was observed to have a relatively high enzyme activity of GPX1 in comparison with the CT or TT variant alleles (Irfan et al. 2016). 

Heme oxygenase-1 (HO-1) represents a protein that is stress-inducible with potent anti-inflammatory and antioxidant properties (Fetoni et al. 2014). It was demonstrated to have high fold alteration in the hair cells of the cochlea. Due to its importance in cell response to stress, triggering of HO-1 protein is demonstrated by multiple oxidative substances or conditions like hyperoxia, heme (Chan Kwon et al. 2020), and hypoxia (Tian et al. 2020). Distribution of HO-1 protein is widely in the kidneys, liver, lungs, and other organs that include the inner ear (He et al. 2019; Yi et al. 2020; Yang et al. 2022).

The aim of the current study is to investigate the association of both hOGG1 and GPx-1 polymorphisms with hearing impairment and HO-1 protein in noisy environment among textile workers.

## Materials and methods

A cross-sectional study was performed on 115 male workers in a spinning and weaving factory located at the Giza governorate. All workers filled out a questionnaire concerning their personal data, detailed history of previous and current jobs, and smoking habits. Hearing problems, any previous ear operation, pus discharge, or histories of chronic drug intake were recorded. Any workers with any previous ear diseases as well as those reporting former intake of ototoxic drug such as aspirin, quinolones, and aminoglycosides were excluded. A general clinical examination was performed, in addition to a local otoscopic examination for the exclusion of any local ear problems. Written informed consent was obtained from all participants. Prior to the beginning of the study, an approval was obtained from the ethical committee at the National Research Centre.

### Audiometry

An audiometric test was performed for 115 subjects who achieved the inclusion criteria, using a manual pure-tone diagnostic audiometer (Model GSI 67, Grason-Stadler, Inc., Eden Prairie, MN, USA). Audiometry was tested on chosen workers in a sound-isolated room that adhered to the American National Standards Institute requirements for an audiometric testing environment. Workers underwent pure-tone audiometry at frequencies of 0.5, 1, 2, 3, 4, 6, and 8 kHz for both ears. The normal hearing definition was known as hearing threshold ≤ 25 dB HL over 0.5–8 kHz frequencies while hearing loss was shown at hearing threshold > 25 dB HL at any frequency.

### Blood pressure measurement

Blood pressure in terms of systolic and diastolic blood pressure was measured under basal conditions using a mercurial sphygmomanometer. According to the International Society of Hypertension and the World Health Organization, high blood pressure is considered ≥ 140/90 mmHg (Chalmers et al. 1999). Under the basal condition, over a week period, multiple readings were taken daily and hypertension diagnosis was done upon taking the average value.

### Sample collection

Four ml of peripheral blood specimens from all study participants were collected into an EDTA vacuum tube (for genotyping of hOGG1 and GPx polymorphism) and a dry tube for serum separation for HO-1 determination.

### Gene assessment

Extraction of DNA from peripheral blood (WBCs) was achieved using a QIAmp extraction kit for genetic analysis of hOGG1 and GPx polymorphisms.

#### Genotyping of hOGG1 polymorphism

According to Musak et al. (2008), the PCR–RFLP-based method was used for analyzing gene encoding DNA repair enzyme (hOGG1) Ser326Cys at exon 7. PCR reaction was produced in 25 µl reaction volume containing 50 ng of genomic DNA, 0.3 mM each primer (forward primer 5′-AGT GGA TTC TCA TTG CCT TCG-3′, reverse primer 5′-GGT GCT TGG GGA ATT TCT TT-3′), 0.2 U Taq DNA polymerase, 20 mM Tris–HCl, 50 mM KCl, 2.0 mM MgCl2, and 0.3 mM each dNTP. PCR program conditions were 30 cycles of denaturation at 94 °C for 30 s, annealing 59 °C for 30 s, elongation at 72 °C for 30 s, and final extension at 72 °C for 5 min. Digestion of the amplified fragments was with restriction endonucleases Fnu4HI. Digested PCR products were separated on 3% agarose gels stained with ethidium bromide and visualized using UV Transilluminator under UV light. Separated hOGG1 was shown as follows: Ser326Cys wild type (WT) genotype, Het heterozygous genotype, Var variant genotype as follows: WT = 250 bp, Het = 250 + 96 + 154 bp, Var = 154 + 96 bp.

#### Genotyping of GPx polymorphism

Genotyping of GPx polymorphism was analyzed, according to Irfan et al. (2016), using one reverse and two forward primers as follows: forward primer 1 (F1) 5′CGCCTGCTGG CCTCCCCTTAC 3′, forward primer 2 (F2) 5′-CGCCTGCTGGCCTCCCCTTAT-3′, and reverse primer (R) 5′-GCAGGGAGCCCAGGCTCACAG-3′ to amplify rs1800668. Primer’s annealing temperature was 62 °C. The size of the PCR product was 172 base pairs and separated on 2% agarose gel stained with ethidium bromide and visualized using UV Transilluminator. In the presence of CC homozygous sample, 172 bp bands appear with reverse primer and F1 primer. In TT homozygous samples, 172 bp bands were shown with F2 primer and reverse primer while CT heterozygous samples, 172 bp bands appear with the reverse primer and both of the two forward F1 and F2 primers.

#### Biochemical analysis of serum heme oxygenase

An enzyme-linked immunosorbent assay (ELISA) commercial kit was used to determine levels of serum hemeoxygenase (SinoGene Clon Biotech Co., Ltd).

### Statistical analysis

Data analysis was done through SPSS program version 18. The expression of results was as mean ± SD. Comparing differences between groups was performed using independent Student’s *t*-test. Significance was set at *P* < 0.05. The *χ*2 test was used to examine genotype distribution between worker groups. Analysis of variance (ANOVA) and post-hoc test of least significant differences (LSD) were used to compare significant differences between different parameters in the 2 genotypes. Multivariable logistic regression analysis was used to explore the relationship between hearing impairment in one ear or in both ears with all factors.

## Results

Table 1 illustrates that the majority of subjects were non-diabetic (91.3%). Most of the workers (76.5%) were nonsmokers. In addition, the absence of audiometric notches was detected in 26% of the workers, while 13% recorded audiometric notches at < 25 dBs and 61% at > 25 dBs. Ear complaints were reported in thirty-six workers (31.3%) in the form of tinnitus and inflammation.

**Table 1:** General characteristic of participants

Parameters	(Mean ± SD)
Age (years)	39.10 ± 5.89
Duration of exposure (years)	17.0 ± 4.95
BMI	27.15 ± 5.04
Systolic pressure (mmHg)	127.96 ± 17.56
Diastolic pressure (mmHg)	81.89 ± 10.82
HO-1 (ng/mL)	7.94 ± 4.0
Diabetes	
Yes	10 (8.7%)
No	105 (91.3%)
Ear complaints	
Yes	36 (31.3%)
No	79 (68.7%)
Smoking	
Yes	27 (23.5%)
No	88 (76.5%)
Notches	
Absent notch	30 (26%)
< 25	15 (13%)
> 25	70 ( 61%)

Also, by analyzing hearing loss in both ears, we found 41 workers (35.7%) showing no hearing impairment, while 52 (45.2%) suffered a mild degree of hearing loss, and 22 (19.1%) showed a moderate loss in the right ear. Moreover, 49 workers (42.6%) had no defect in hearing in the left ear, while 45 (39.5%) and 21 (18.2%) showed mild and moderate hearing loss, respectively, in the left ear (Table 2).

**Table 2:** Hearing impairment among studied workers

Hearing impairment	Right ear no. (%)	Left ear no. (%)
Normal (0–25 dB)	41(35.7%)	49 (42.6%)
Mild (26–40 dB)	52(45.2%)	45(39.1%)
Moderate (41–60 dB)	22(19.1%)	21(18.2%)

**Fig. 1:**
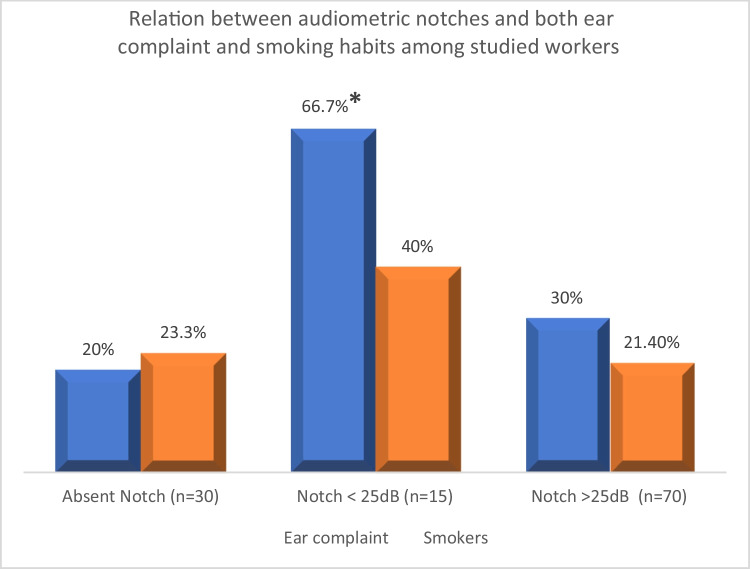
Relation between audiometric notches and both ear complaint and smoking habits among studied workers

Figure 1 demonstrates high significant change between the existence of audiometric notches and ear complaints (*P* = 0.012). The workers with ear complaint represented 20% for those with an absent notch, 66.7% with audiometric notch < 25 dBs, and 30% with audiometric notch at > 25 dBs. Also, 80% without ear complaint had no notch, 33.3% showed a notch at < 25, and 70% with audiometric notch at > 25 dBs. There was no relation between smoking and the presence of audiometric notches in the studied workers (*P* = 0.5).

**Fig. 2:**
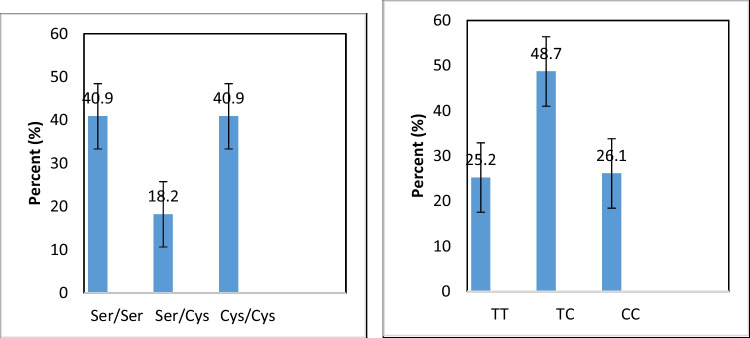
Frequency distribution of hOGG1 and GPx polymorphisms among studied workers

Figure 2 demonstrates the frequency distribution of hOGG1 and GPx polymorphisms among textile workers. Homozygous Ser/Ser polymorphism of hOGG1 represented 40.9% (47 workers), heterozygous Ser/Cys 18.2% (21 workers), and mutant Cys/Cys 40.9% (47 workers). In GPx polymorphism, homozygous TT polymorphism was detected in 25.2% (29 workers), heterozygous TC in 48.7% (56 workers), and mutant CC polymorphism in 26.1% (30 workers). No significant frequency distribution was shown in both hOGG1 and GPx polymorphisms.

**Fig. 3:**
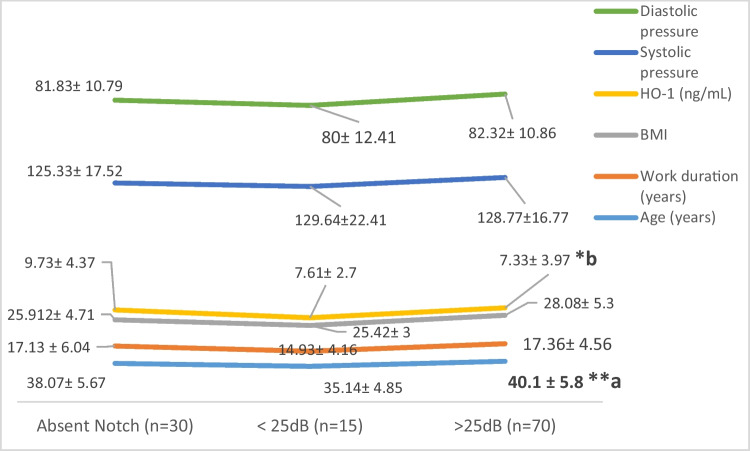
Comparison between presence of audiometric notches and different parameters among studied workers

Figure 3 illustrates that audiometric notches > 25 dB were present in older textile workers with a high significant difference (*P* < 0.009) compared to lower aged ones, as well as significant difference (*P* < 0.049) was shown in HO-1 levels showing higher levels among workers with the absence of notch.

**Fig. 4:**
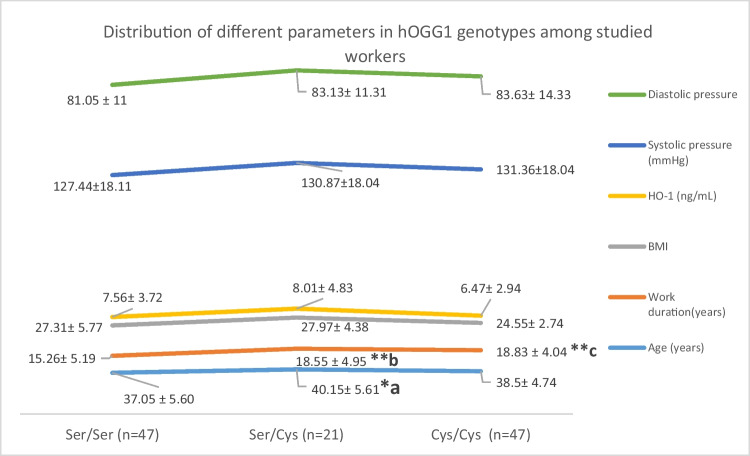
Distribution of different parameters in hOGG1 genotypes among studied workers

Figure 4 demonstrates that age showed a significant difference (*P* < 0.042) between different hOGG1 genotypes among textile workers showing heterozygous genotype Ser/Cys in older aged workers, as well as high significant difference (*P* = 0.006) was shown with work duration. Mutant genotypes Cys/Cys were shown in workers with increased work duration.

**Fig. 5:**
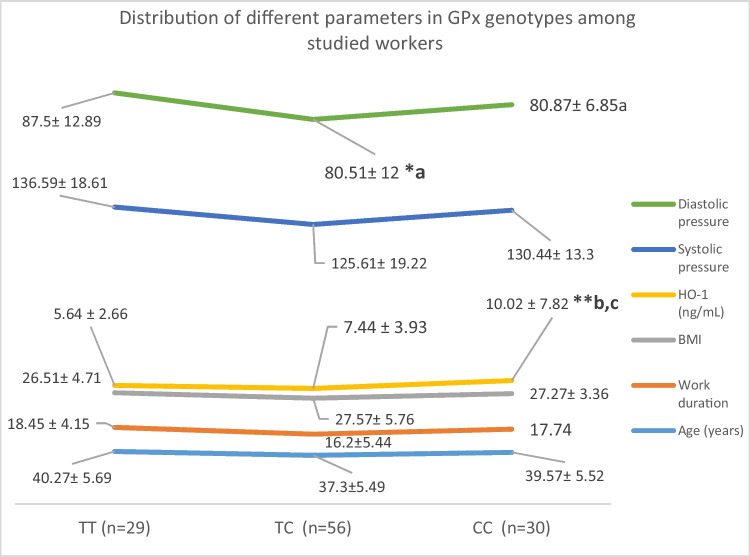
Distribution of different parameters in GPx genotypes among studied workers

Figure 5 revealed high significant difference (*P* < 0.001) among textile workers in HO-1 levels between different GPx genotypes, and CC (mutant) genotypes were associated with higher HO-1 levels followed by TC (heterozygote) genotype. Also, a significant difference (*P* < 0.05) was shown in diastolic pressure between different GPx genotypes with higher levels in TT genotype.

**Fig. 6:**
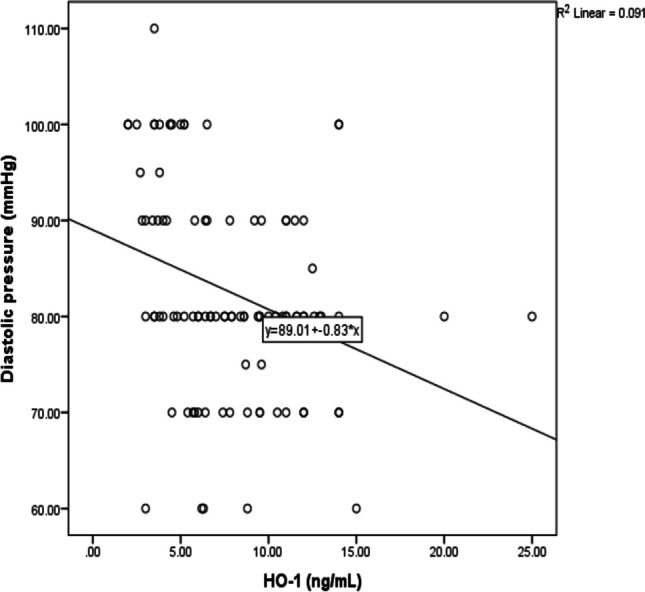
Correlation between diastolic pressure and HO-1 protein among textile workers

**Fig. 7:**
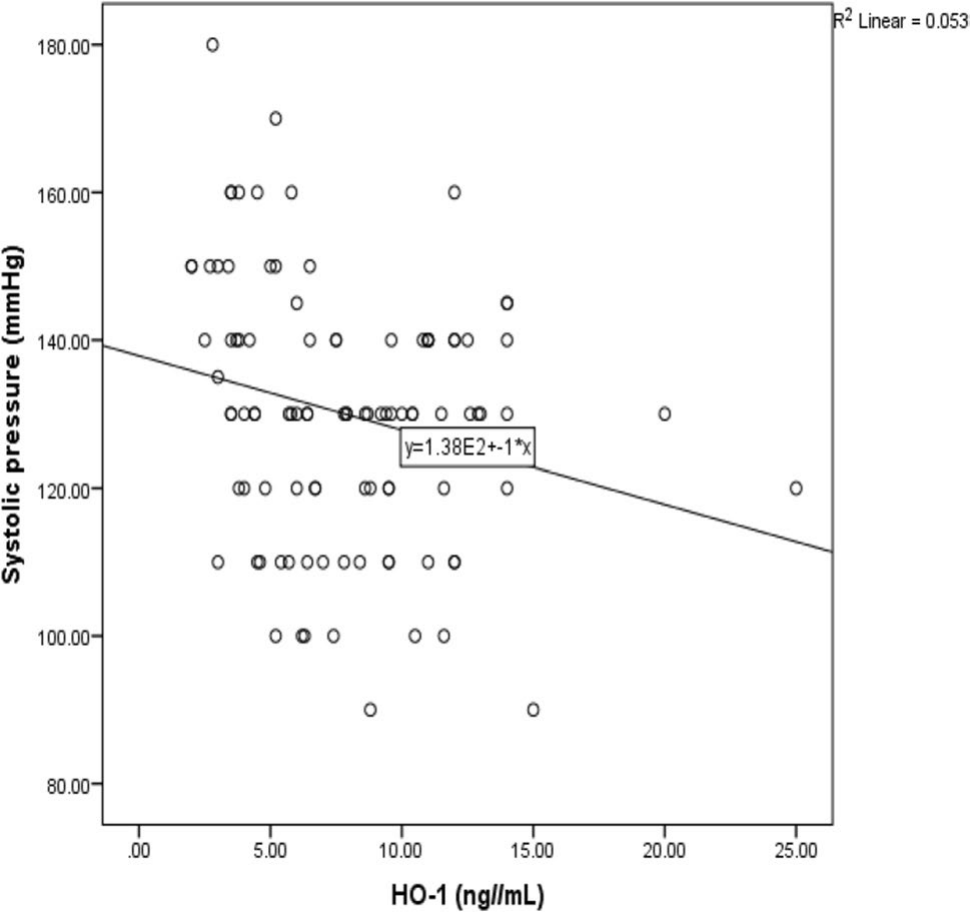
Correlation between systolic pressure and HO-1 protein among textile workers

Figure 6 demonstrated negative correlation between diastolic pressure and HO-1 protein among textile workers occupationally exposed to noise (*r* =  − 0.3, *P* < 0.002) as well as a negative correlation between systolic pressure and HO-1 protein (*r* =  − 0.23, *P* < 0.022) as shown in Fig. 7.

Table 3 illustrates logistic regression for the factors affecting the occurrence of hearing impairment in the studied group. Factors such as diabetes (OR 5% with 95% C.I (0.003–0.8)), longer exposure duration [OR 64% with 95% C.I (50.4–81.5%)], older age [OR 1.46 and 95% C.I (1.18–1.8)] and TC genotype of GPx gene [OR18% and 95% C.I (4–80%)] were associated with hearing impairment.

**Table 3:** Variables affecting hearing impairment among textile group

Explanatory variables		*P* value	OR	95% CI lower	95% CI upper
Positive smoking	0.044	0.83	0.86	0.21	3.52
Work duration (yrs)	13.15	0.001*	0.64	0.5	0.81
Age	12.69	0.001*	1.46	1.19	1.8
HO-1 (ng/mL)	1.05	0.31	0.93	0.8	1.07
hOGG	2.66	0.26			
hOGG					
Ser/Ser	0.11	0.74	1.24	0.36	4.31
hOGG					
Ser/Cys	1.85	0.17	0.3	0.053	1.7
GPx	6.33	0.042*			
GPx					
TT	0.064	0.80	0.81	0.15	4.28
GPx					
TC	5.1	0.024*	0.19	0.044	0.80

## Discussion

Chronic occupational exposure to noise is an unavoidable reality in the textile industry in different countries (Kheirandish and Bidel 2021). NIHL is the most common work-related disease that can gradually produce progressive impairment as well as a disturbance in patient’s quality of life (Pretzsch et al. 2021).

The current study recorded high significant relation between audiometric notches and ear complaints (*P* < 0.012), and according to Damayanti et al. (2022), human characteristics showed weak to moderate relationship with subjected hearing complaints. In other words, a strong relationship was recorded between exposure to noise intensity and subjected hearing complaints. Also, older age workers showed audiometric notches at > 25 dB. Significant differences in HO-1 levels (*P* < 0.049) with higher levels among workers with normal audiometry can be attributed to the functional effect of HO-1 induction. In turn, HO-1 induction reflects cell ability to resist injuries resulted from multiple stresses with over- or under-expressed HO-1. Furthermore, it reflects the critical defense effect of HO-1 cell on oxidative stress (Yang et al. 2022). Also, previous study reported that HO-1 can act as an additional mediator, in vivo and in vitro, against damage resulted upon noise or ototoxic drug which can affect cochlea. Moreover, HO-mediated carbon monoxide generation had the ability to protect against immune-mediated diseases as well as vascular dysfunction, in addition to its essential role in modulating tympanogenic cochlear inflammation (Bing et al. 2019).

NIHL pathophysiology was attributed to multifactorial and complex factors such as genetic and environmental one with substantial contributions of occupation (Natarajan et al. 2023). Our study found no significant frequency distribution in both hOGG1 and GPx polymorphisms. In the base excision repair (BER) pathway, human hOGG1 is a key enzyme that contributes in 8-oxoG serum bilirubin level elimination (serum bilirubin represents here a potent marker for the hearing outcome in severe-profound bilateral sudden deafness) (Shen et al. 2014). The present study demonstrated that heterozygote Ser/Cys genotype of hOGG1 gene was shown in older age workers while workers with longer work duration were shown to have the mutant genotype Cys/Cys. Chen et al. (2022) mentioned that, in the Chinese Han population, hOGG1 Cys/Cys genotype may represent a common risk factor for hearing loss at high frequency. Stratified analysis recorded an association between this genotype and other risk factors like duration of work (in years) in that noisy job and noise exposure level.

One of the five GPx homologues, GPx-1, represents the most distributed enzyme widely and especially in vascular endothelial cells (Forgione et al. 2002). It can protect superiorly against SOD and CAT in cells exposed to ROS (Shao et al. 2020).

Finally, a high significant change in HO-1 levels was reported between different GPx genotypes among textile workers. Mutant CC genotype was associated with high levels in HO-1 followed by TC (heterozygote) genotyp. Irfan et al. (2016) suggested that GPX-1 gene polymorphism may be an essential factor of the gene susceptibility for NIHL.

Differences in enzymatic activity that resulted from genotype can disturb the delicate balance between minimizing the oxidative stress and clearance of ROS. In antioxidant genes, increased levels of ROS and reduced enzymatic activity may occur due to functional polymorphisms that may confirm its role in contributing towards increasing risk of age-related disorders (Irfan et al. 2016). Previous study conducted by Shao et al. (2020) revealed that especially rs1800668 GPx-1 polymorphisms may affect GPx-1 promoter activity. In addition, C allele was shown to be associated with an increase in GPx activity which can help in improving antioxidant activity. In addition, wild-type TT genotype was associated with high diastolic pressure. A recent study concluded that the level of sound pressure that is over 85 dB had mainly acute effects and psychological and physical damage. Only this sound level can lead to an increased risk of hypertension as well as otherwise cardiovascular diseases (Alimohammadi et al. 2020). HO-1 protects against inflammatory and oxidative insults in case of hypertension in order to reduce blood pressure and end-organ damage through its expression at the vascular level and to shift macrophages toward anti-inflammatory phenotype.

Finally, logistic regression conducted revealed that the presence or absence of t notch is independent on the following factors such as smoking, older age, or presence of genetic deviation (data not shown). While hearing impairment was linked to longer exposure duration to noise as well as older age and the presence of heterozygote genotype (TC) of GPx gene in the studied textile workers.

Regarding other antioxidant gene like CAT and SOD genes and its associations with NIHL, a meta-analysis conducted by Wu et al. (2023) demonstrated a significant relationship between CAT polymorphism (rs208679) and NIHL in the allele model (A vs. G) and dominant model (AA vs. GG + AG), where the A allele is mutated to G allele, could affect transcription factor binding and as a consequence, CAT enzymatic activity decreases and not the expression level that could result in an increase in hydroxyl radicals’ formation and elevated diseases risk.

Another study conducted on 225 healthy volunteers and 494 workers exposed to a varying noise intensity showed that CAT gene (rs208679), the GG genotype (recessive effect), was significantly associated to an augmented risk when individuals are exposed to noise less than 85 dB; however, (for rs769217 dominant effect), the TT/TC combined genotypes were in association with risk of NIHL when exposed to noise intensity between 85 and 92 dB (Yang et al. 2015).

Another review article conducted by Chen et al. (2022) on Chinese Han people (*n* = 2400), occupationally exposed to noise, recorded that the SOD1 AA genotype (rs2070424) was protective against NIHL, while (rs10432782) the SOD1 GG genotype and (rs4880) CT genotype of (SOD2 V16A SNP) was in association with higher occurrence of NIHL.

## Limitation of the study

As shown in our study, no control group of non-occupationally noise-exposed workers was included. So, we cannot propose conclusions comparing workers with normal control subjects. Another limitation was shown in the small sample size. Further studies with larger sample size had to be conducted with the control group to reach definite conclusions.

## Conclusion

In textile factories, a high incidence for NIHL was observed present among workers that indicate the mandatory and essential use of different protective measures. NIHL diagnosis is based on several factors which predict likelihood damage of inner ear resulting from excessive exposure to noise. Heterozygote Ser/Cys genotype of hOGG1 gene was shown in older age workers while mutant Cys/Cys genotype was associated with longer work duration in textile workers. Mutant CC genotype of GPx gene was associated with higher levels of HO-1 followed by TC genotype, and wild type TT genotype was associated with higher diastolic pressure levels. Finally, hearing impairment was shown to be dependent on the duration of exposure to noise in addition to older age and the presence of heterozygote TC genotype of GPx gene.

## Recommendations

In the textile industry hearing conservation programs are warranted strongly. Also, to all workers, hearing protective equipment must be available with a follow-up regularly and especially for whom audiograms showing the presence of notches at their periodic hearing tests. In addition, hearing screenings may reduce any delays in the diagnosis as well as provision of hearing aids for those suffering from hearing loss, leading to an improvement in their health-related quality of life.

## Data Availability

Sharing data for this article is not applicable as no datasets were generated during present study.
